# Does Lead Have a Connection to Autism? A Systematic Review and Meta-Analysis

**DOI:** 10.3390/toxics11090753

**Published:** 2023-09-05

**Authors:** Aleksandar Stojsavljević, Novak Lakićević, Slađan Pavlović

**Affiliations:** 1 Innovative Centre, Faculty of Chemistry, University of Belgrade, Studentski Trg 12–16, 11000 Belgrade, Serbia; 2 Clinical Centre of Montenegro, Clinic for Neurosurgery, Ljubljanska bb, 81000 Podgorica, Montenegro; novak.lakicevic@kccg.me; 3 Institute for Biological Research “Siniša Stanković”—National Institute of the Republic of Serbia, University of Belgrade, Bulevar Despota Stefana 142, 11060 Belgrade, Serbia; sladjan@ibiss.bg.ac.rs

**Keywords:** lead, autism, biological material, systematic review, meta-analysis

## Abstract

Environmental pollutants, particularly toxic trace metals with neurotoxic potential, have been related to the genesis of autism. One of these metals that stands out, in particular, is lead (Pb). We conducted an in-depth systematic review and meta-analysis of peer-reviewed studies on Pb levels in biological materials retrieved from autistic children (cases) and neurotypical children (controls) in this work. A systematic review was conducted after the careful selection of published studies according to established criteria to gain a broad insight into the higher or lower levels of Pb in the biological materials of cases and controls, and the findings were then strengthened by a meta-analysis. The meta-analysis included 17 studies (hair), 13 studies (whole blood), and 8 studies (urine). The overall number of controls/cases was 869/915 (hair), 670/755 (whole blood), and 344/373 (urine). This meta-analysis showed significantly higher Pb levels in all three types of biological material in cases than in controls, suggesting a higher body Pb burden in autistic children. Thus, environmental Pb exposure could be related to the genesis of autism. Since no level of Pb can be considered safe, the data from this study undoubtedly point to the importance of regularly monitoring Pb levels in autistic children.

## 1. Introduction

Autism is a complex neurodevelopmental disturbance primarily characterized by difficulties in social communication and interaction, together with restricted and/or repetitive behaviors or interests [1,2]. The incidence of autism has increased substantially over the past few decades [3,4]. According to the most recent World Health Organization (WHO) data, one in every 100 children globally is autistic [5]. According to a 2020 Centers for Disease Control and Prevention (CDC) report, one in every 36 children in the United States is autistic [6]. It affects 1–2% of all children globally, with the majority of cases identified in affluent countries [7]. Autism is approximately four times more common in boys than in girls [8,9,10]. Since there is no reliable biomarker for early diagnosis, extraordinary efforts are underway to uncover the underlying mechanisms for the neurodevelopmental differences common to autistic people [1].

The etiology of autism is most likely multifactorial; a genetic component and environmental triggers have been suggested in the development of autism [11]. Among the environmental triggers, trace metals receive special attention due to their neurotoxic effects and unique neurochemistry [12,13].

Toxic trace elements are an integral part of the environment, although accelerated and uncontrolled industrialization has dramatically increased their levels in the last 50 years [14,15]. Not surprisingly, biomonitoring studies have indicated different elemental profiles across various populations around the world, suggesting that the extent of human exposure to trace metals directly depends on where one lives [16,17,18]. Unacceptably high levels of children’s exposure to toxic trace metals have been documented. Their harmful effects are reflected in reproductive and developmental health, as toxic trace metals can cause fetal malformations, developmental delays, neurological diseases, learning difficulties, and behavioral abnormalities [19].

Lead (Pb) is one of the most extensively researched trace elements in autism. It is a heavy, non-essential metal and non-threshold neurotoxicant [17,20]. Although Pb is naturally present in the environment, today’s considerably high Pb levels in the environment are a direct consequence of the industrial revolution and rapid urbanization [18,21,22,23]. Despite national and international efforts to reduce Pb levels in the environment, it remains one of the greatest pollutants [24,25,26]. Thus, high Pb levels are consistently recorded in cities and developed industrial areas [27].

Lead exposure in children has been a long-standing public health issue in many countries. It is primarily taken into the body by ingestion and inhalation [28,29]. Lead-containing paints (primarily yellow pigments in paints), leaded gasoline, dust, tin toys, Pb-contaminated food, and drinking water are major sources of this non-essential metal and pose a serious hazard to developing children [3]. Given their innate curiosity and inability to distinguish non-food items from actual food, young children frequently exhibit a persistent craving for non-food items. Therefore, children are more susceptible to Pb exposure than adults due to higher oral intake and absorption rates, particularly if they lack essential elements [30]. Compared to adults, who absorb 1–10% of Pb by ingestion, children absorb approximately five times more Pb via the gastrointestinal tract [31]. However, it is important to emphasize that Pb exposure begins prenatally and continues throughout life. Lead can easily cross biological barriers (placental and blood–brain barrier) and accumulates in internal organs, primarily in bones and lipid-dense organs, including the brain [32]. Erythrocyte turnover of Pb occurs within approximately 120 days. Lead is ultimately excreted in bile or urine [33].

Lead has a strong and irreversible effect on children’s health; it is a powerful initiator or promoter of multiple diseases, even at small levels [30]. Young children’s nervous systems are more sensitive to Pb exposure than adults’, making their brains more susceptible to disturbances in central neurological functions, such as developmental delays, gross and fine motor deficits, social withdrawal, hyperactivity, intellectual and behavioral deficits, and a reduced intelligence quotient (IQ) [34]. In 2012, the CDC recommended lowering the allowable blood Pb level in children from the initial 10 µg/dL to half this amount. Regardless of the blood Pb threshold, studies around the world warn that no level of Pb in children can be deemed safe [30,35]. Thus, Pb is considered a neurotoxin with an “unsafe” level [36].

## 2. Materials and Methods

The “Preferred Reporting Items for Systematic Reviews and Meta-Analyses: The PRISMA Statement” [37] was followed for this systematic review and meta-analysis. The PRISMA statement includes a 27-item checklist to help authors to perform systematic reviews and meta-analyses. The authors established the research protocol before beginning the current trial.

### 2.1. Information Sources

For this study, four databases were initially examined: PubMed, ScienceDirect, SCOPUS, and Google Scholar. Due to the overlap of publications from these databases in a relevant percentage, we selected two of the most representative databases for the search, PubMed and ScienceDirect.

### 2.2. Search Strategy

**Figure 1:**
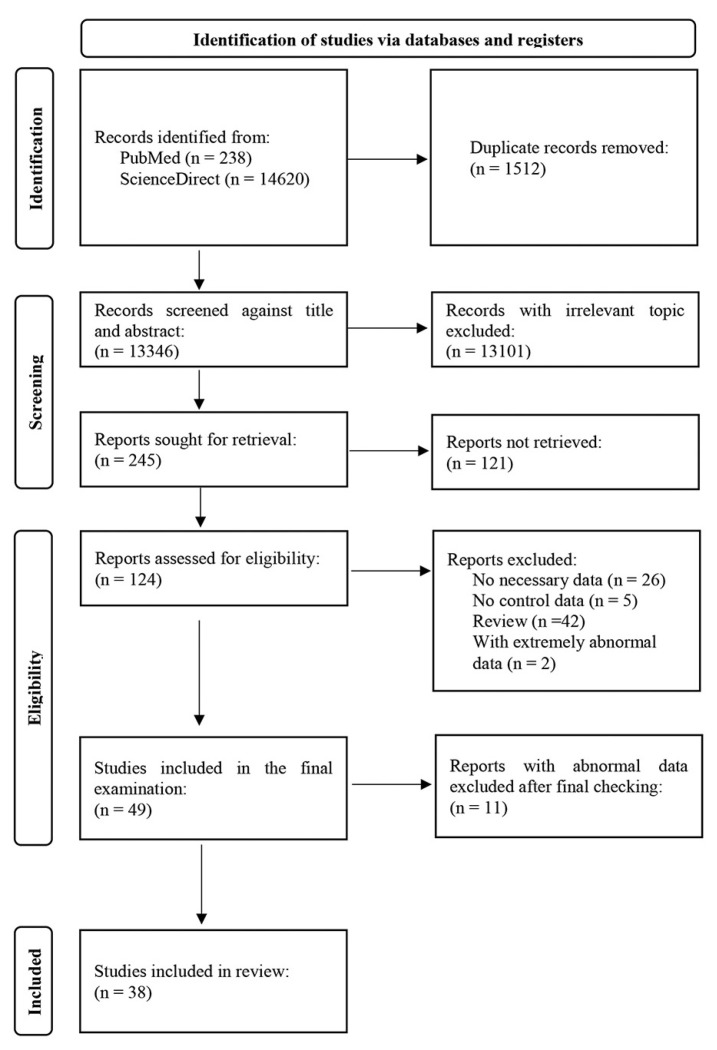
PRISMA flow diagram of literature search and study identification, screening, eligibility, inclusion, and exclusion. Combined data for hair, whole blood, and urine.

The primary purpose of this study was to identify all papers that examined Pb levels in the hair, whole blood, serum/plasma, red blood cells (RBCs), urine, and teeth collected from autistic children (cases) and non-autistic people (children without identified disabilities, neurotypical children—controls). The literature search encompassed 2005 to the present. We used the following Mesh terms in the search: “autism” AND “autistic” AND “autism spectrum disorder” AND “child” AND “preschool” AND “school” AND “adolescent” AND “heavy metals” AND “toxic metals” AND “lead” AND “Pb” AND “hair” AND “blood” AND “serum/plasma” AND “red blood cells” AND “urine” AND “teeth”. We also screened the reference lists of retrieved results. Inclusion criteria were original research studies reporting Pb levels in the mentioned biological materials from cases and controls. Exclusion criteria were studies that considered adults, studies in which the diagnosis of autism was not confirmed, studies in which cases and controls were not from the same place of residence, studies in which additional pathology was reported in addition to autism, studies not written in English, studies that did not offer sufficient numerical data, and studies with extremely abnormal Pb values. Additional exclusion factors are listed in Figure 1. For the meta-analysis, we examined full-length original research articles from 2005–2023 for hair, 2011–2023 for whole blood, and 2010–2020 for urine Pb levels. These time periods were chosen to make the analytical procedures as consistent as possible. Most authors used inductively coupled plasma mass spectrometry (ICP-MS) to determine Pb concentrations in biological materials, while a smaller number of papers used atomic absorption spectroscopy (AAS). Only one paper reported the energy-dispersive X-ray (EDX) technique.

### 2.3. Study Selection and Data Extraction

PRISMA procedures were followed in the selection of papers, which included identification, screening, eligibility, and inclusion. This paper selection process is graphically illustrated in Figure 1. Two trained researchers (A.S. and S.P.) independently extracted the following data: author(s) and year of publication, country, sample size (controls/cases), age (controls/cases), sex (number of girls/boys in both groups, presented as controls/cases), type of biological material, analytical technique, and Pb level (mean ± SD, presented as controls/cases). In papers where results were reported as the mean ± SEM, the SEM was converted to the SD using the appropriate formula. If authors presented results as an interquartile range (IQR), then the IQR was also converted to the SD [38]. We only considered papers that explicitly presented their results as numerical values. At the end of selection and data extraction, the final list was prepared by consensus.

### 2.4. Quality Assessment

Quality assessment of the included studies was performed using the Newcastle–Ottawa scale (NOS) according to [39]. Quality assessment was performed using the modified criteria of [40]. Possible scores ranged from 1 to 7, and studies that scored 7 were considered to be of the highest quality and at the lowest risk of bias. Studies that scored below 7 were considered to be of lower quality and at a higher risk of bias.

### 2.5. Statistical Analysis

The heterogeneity of the selected studies was estimated using the I-squared (I^2^) and the associated Cochran’s Q test [41]. An I^2^ value of 75% was considered a high level of heterogeneity, and because of the low power of the Q test to detect heterogeneity, *p* < 0.1 was considered significant. When heterogeneity was more than 70%, the pooled estimates were analyzed using the random-effects model. All analyses were conducted using the random-effects model, since we expected the true effect sizes to vary across studies. According to [42], τ-squared (τ^2^) also indicates heterogeneity. τ^2^ close to 0 indicates low heterogeneity and τ^2^ greater than 1 indicates substantial heterogeneity [43]. The effect size was calculated as the mean differences in Pb levels in biological material (hair, whole blood, and urine) and converted into Hedges’s g, which allows adjustment for influences of the small sample sizes [44]. A 95% confidence interval (CI) was calculated to examine the statistical discrepancy of the pooled size effects. We also calculated the relative weight of the study, which provided information on the contribution of each study to the overall summary results of our meta-analysis. This was particularly important for those studies that were considered as outliers or had a high risk of bias. The standard residual was also estimated and represented the unaccounted-for remaining variability between studies. *p* values less than two-sided 0.05 were considered significant. Statistical analysis was performed using Comprehensive Meta-Analysis software (v. 3.0, Biostat Inc., Frederick, MD, USA).

### 2.6. Publication Bias

Publication bias was assessed using Egger’s regression test [45] and Begg and Mazumdar’s rank correlation test [46]. For each type of biological material (hair, whole blood, and urine), publication bias was visually illustrated using funnel plots.

In determining the publication bias, the fail-safe method was also used for all three types of biological materials, to determine the number of missing studies that should be added to improve the quality of the meta-analysis, but these results are not presented here. We considered that they did not significantly affect the quality of the obtained results, which, in accordance with the studies analyzed, showed significantly higher Pb levels in the biological materials of cases compared to controls.

## 3. Results

### 3.1. Study Selection and Identification

The PRISMA flow diagram of the literature search and study identification, screening, eligibility, inclusion, and exclusion is presented in Figure 1. The diagram includes combined literature data for hair, whole blood, and urine. The agreement specified that the two most relevant databases, PubMed and ScienceDirect, would be searched. Our initial search resulted in 14,858 records. After removing 1512 duplicates, we selected 13,346 reports against the title and abstract. After this, we excluded 13,101 reports with irrelevant topics. The remaining 245 reports were sought for retrieval. Altogether, 121 reports were not retrieved, and the remaining 124 reports were evaluated for eligibility. From these 124 reports, 26 without necessary data, 5 reports without control data, 42 review reports, and 2 reports with extremely abnormal data were all excluded (total excluded, n = 75 reports). The final examination included 49 studies. After an additional check, we excluded 11 of these studies with abnormal data. Finally, our meta-analysis included 38 studies. From these studies, 17 studies considered Pb levels in hair, 13 in whole blood, and 8 in urine. The time frame included studies from 2005–2023 for hair, 2010–2023 for whole blood, and 2010–2020 for urine Pb levels. The total number of control participants was 1883, while the total number of cases was 2043.

### 3.2. Study Characteristics

The characteristics of the studies included in the meta-analysis are shown in Table 1. The meta-analysis of Pb levels in hair included 17 studies [19,32,47,48,49,50,51,52,53,54,55,56,57,58,59,60,61], 13 studies in whole blood [20,31,34,44,58,62,63,64,65,66,67,68,69], and 8 studies in urine [24,34,36,58,70,71,72,73,74].

Regarding Pb levels in hair, four studies were conducted in Europe [32,49,53,58], two studies in North America [54,60], eight studies in Asia [19,47,48,51,55,56,59,61], and three studies in Africa [50,52,57]. Regarding Pb levels in whole blood, two studies were carried out in Europe [58,62], two studies in North America [34,64], one study in Central America [65], five studies in Asia [31,44,63,68,69], and three studies in Africa [20,66,67]. Our meta-analysis of urine Pb levels included data from three European studies [24,58,71], two studies from North America [34,70], and three studies from Asia [36,73,74].

### 3.3. Quality Assessment

Quality scores ranged from 1 to 7, with an average score of 6.57. The study quality score for hair studies was 6.35, for whole blood studies 6.62, and for urine studies 6.75 (Table 2). The scores of 5 and 6 that we assigned to certain studies can generally be explained as follows: a score of 5 was assigned to studies that did not represent the sex and age of participants or were missing numerical data (such as standard deviation or error). A score of 6 was assigned to studies that did not report one of the two items from the criteria for a score of 5 (for example, data for participants’ sex were missing, but all necessary numerical data for the meta-analysis were presented). The assigned scores did not indicate lower quality of the selected studies, but indicated the non-fulfillment of the criteria that we set in this meta-analysis.

### 3.4. Meta-Analysis of Pb Levels in Hair

This part of the analysis included 17 studies with a sample size of 869 controls and 915 cases (Table 1). In the two studies, ages ranged between 3 and 14 years for controls and 4 and 12 years for cases. For the remaining 15 studies, the mean age was 6.26 years for controls and 6.48 years for cases. In four studies, sex was not reported, and in one study, only boys were investigated. The other 12 studies included 187 girls and 395 boys in the control group and 142 girls and 581 boys in the case group. Eleven studies used ICP-MS as an analytical technique, five studies AAS, and one study used EDX. The mean hair Pb level ranged from 0.01 ± 0.01 µg/g [57] to 6.31 ± 3.74 µg/g [61] for controls, and between 0.07 ± 0.01 µg/g [57] and 19.95 ± 17.23 µg/g [61] for cases. In nine studies, the mean hair Pb level of cases was significantly higher compared to controls; in two studies, it was significantly lower compared to controls; and in six studies, no significant differences were reported (Table 3).

**Figure 2:**
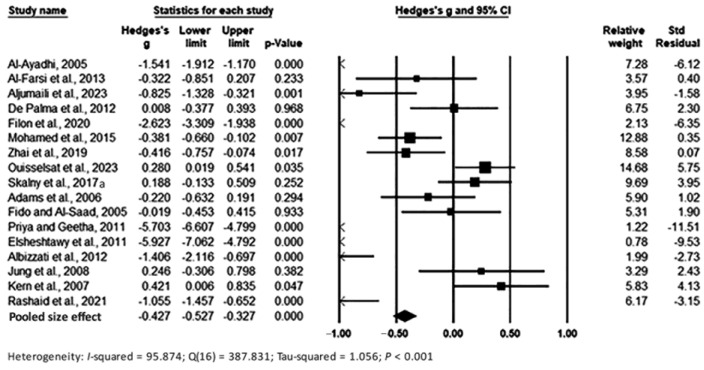
Forest plot for random-effects meta-analysis. Differences in Pb levels in hair samples between controls and cases are shown. The size of each square is proportional to the weight of the study. Based on the observed studies, the diamond symbol indicates the pooled overall effect size. Abbreviation: CI, confidence interval.

Pooling data under the random-effects model showed significant differences between the two groups (cases and controls), with Hedges’s g = −0.427 (95% CI: −0.527, −0.327) and *p* < 0.001, with the effect size in individual studies ranging from −5.927 (95% CI: −7.062, −4.792, *p* < 0.001) [57] to 0.421 (95% CI: 0.006, 0.835, *p* < 0.001) [60] (Figure 2). Relative weights and standard residuals for each study are shown in Figure 2. Relative weights ranged from 0.78% [57] to 14.68% [52]. Standard residuals ranged from −11.51 [56] to 5.75 [52]. Heterogeneity was I^2^ = 95.874%, Q_16_ = 387.831, and τ^2^ = 1.056, *p* < 0.001, indicating high heterogeneity between the true mean effects.

**Figure 3:**
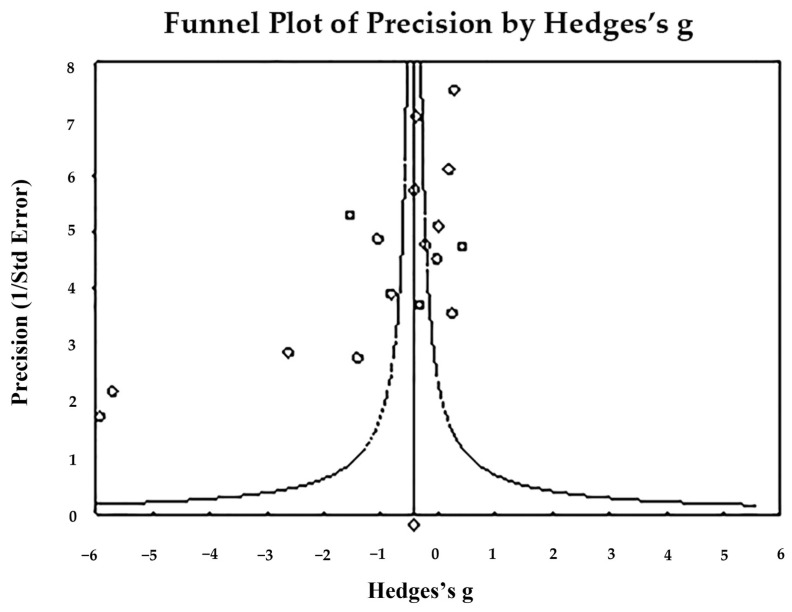
Funnel plot for assessment of publication bias in in observational studies comparing hair Pb levels of controls and cases. The figure shows the studies’ effect sizes (Hedges’s g) relative to their precision (inverse of SE). Circles represent observed studies. Based on the observed studies, the diamond symbol indicates the pooled overall effect size. Publication Bias—Egger’s regression test: t_17_ = 3.972, *p* = 0.001. Begg and Mazumdar rank correlation—Kendall’s τ = −0.389, *p* = 0.029.

The funnel plot (Figure 3) indicates publication bias—Egger’s regression test: t_17_ = 3.972, *p* = 0.001; and Begg and Mazumdar rank correlation: Kendall’s τ = −0.389, *p* = 0.029. In summary, the pooling size effect shows significantly higher hair Pb levels in cases than in controls (*p* < 0.001).

### 3.5. Meta-Analysis of Pb Levels in Whole Blood

The meta-analysis of whole blood Pb levels in controls and cases included 13 studies with a sample size of 670 neurotypical children and 755 autistic children (Table 1). In two studies, age ranged between 2 and 8 years for controls and 6 and 12 years for cases, and in one, it was 4.02 ± 4.01 years for both sexes. In the remaining ten studies, the mean age was 7.24 years for controls and 6.84 years for cases. In two studies, sex was not reported, whereas, in the other 11 studies, 177 girls and 368 boys were included in the control group, while 140 girls and 490 boys were included in the case group. ICP-MS was used in eight studies and AAS in five studies. The mean Pb level ranged from 1.59 ± 1.69 [20] to 58.83 ± 18.92 µg/L [63] in the controls, and from 2.24 ± 3.15 [20] to 94.90 ± 20.20 µg/L [67] in the cases. Blood Pb levels in cases were significantly higher than in controls in three studies, while no significant differences were observed in ten studies (Table 3).

**Figure 4:**
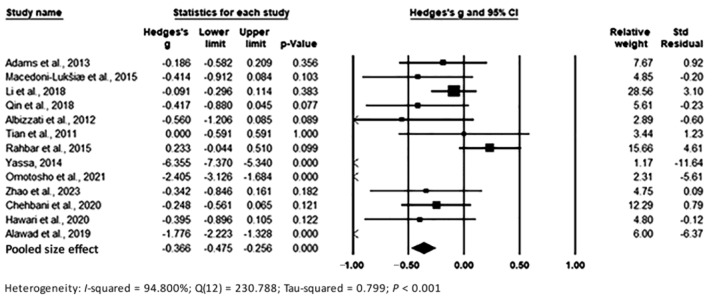
Forest plot for random-effects meta-analysis. Differences in Pb levels in whole blood samples between controls and cases are shown. The size of each square is proportional to the weight of the study. Based on the observed studies, the diamond symbol indicates the pooled overall effect size. Abbreviation: CI, confidence interval.

The pooling of data under the random-effects model is presented in Figure 4. The results show significant differences between the two groups, with Hedges’s g = −0.366 (95% CI: −0.475, −0.256) and *p* < 0.001, with effect sizes in individual studies ranging from −6.355 (95% CI: −7.370, −5.340, *p* < 0.001) [66] to 0.233 (95% CI: −0.044, 0.510, *p* < 0.001) [65]. Relative weights and standard residuals for each study are also shown in Figure 4. Relative weights ranged from 1.17% [66] to 28.56% [63]. Standard residuals ranged from −11.64 [66] to 4.610 [65]. Heterogeneity was I^2^ = 94.800%, Q_12_ = 230.788, and τ^2^ = 0.799, *p* < 0.001, which indicated high heterogeneity in whole blood and which was intermediate regarding the heterogeneity that we found in hair and urine.

**Figure 5:**
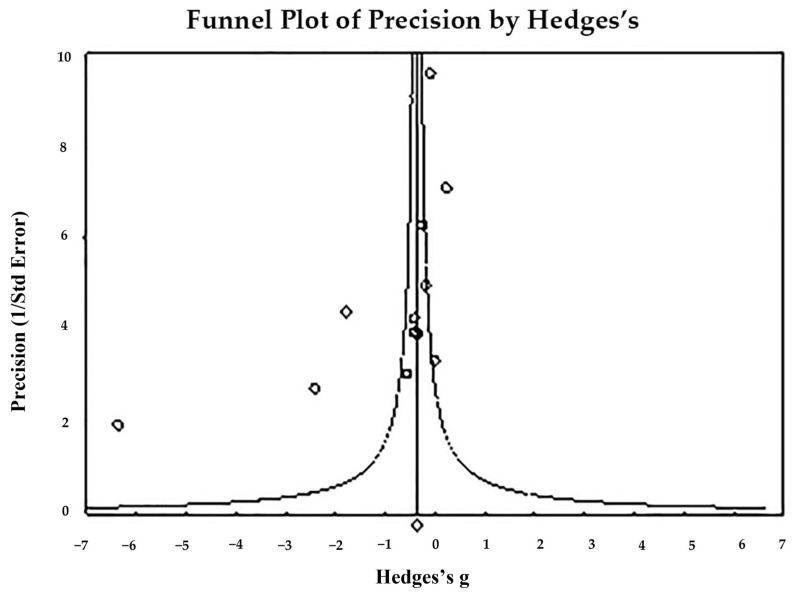
Funnel plot for assessment of publication bias in observational studies comparing whole blood Pb levels of controls and cases. The figure shows the studies’ effect sizes (Hedges’s g) relative to their precision (inverse of SE). Circles represent observed studies. Based on the observed studies, the diamond symbol indicates the pooled overall effect size. Publication Bias—Egger’s regression test: t_13_ = 3.040, *p* = 0.011. Begg and Mazumdar rank correlation: Kendall’s τ = −0.461, *p* = 0.033.

The funnel plot (Figure 5) indicates publication bias—Egger’s regression test: t_13_ = 3.040, *p* = 0.011; and Begg and Mazumdar rank correlation: Kendall’s τ = −0.461, *p* = 0.033. In conclusion, the pooling size effect shows significantly higher whole blood Pb levels in cases than in controls (*p* < 0.001).

### 3.6. Meta-Analysis of Pb Levels in Urine

The meta-analysis of urine Pb levels in controls and cases included eight studies with a sample size of 344 neurotypical children and 373 autistic children (Table 1). In two studies, the mean age was 4.02 ± 4.01 and 9.80 ± 3.30 years for the controls and cases, respectively. In the remaining six studies, the mean age was 6.64 years for the control group and 6.04 years for the case group. The control group included 83 girls and 262 boys, and the case group included 74 girls and 299 boys. ICP-MS was used in seven studies, while AAS was used in one study. The mean Pb level ranged from 0.32 ± 0.45 [34] to 4.63 ± 3.83 µg/g creatinine [71] in the control group, and from 0.57 ± 0.86 [34] to 12.47 ± 17.46 µg/g creatinine [74] in the case group. Urine Pb levels in cases were significantly higher than in controls in four studies, significantly lower in two studies, and without significant differences in two studies (Table 3).

**Figure 6:**
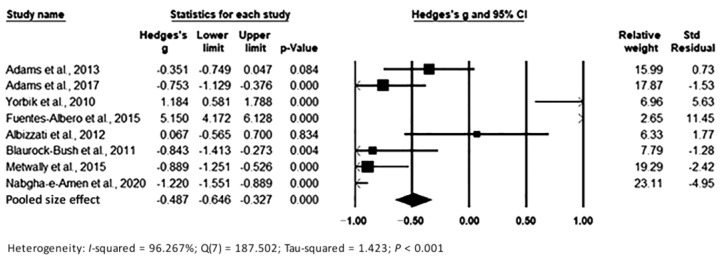
Forest plot for random-effects meta-analysis. Differences in Pb levels in urine samples between controls and cases are shown. The size of each square is proportional to the weight of the study. Based on the observed studies, the diamond symbol indicates the pooled overall effect size. Abbreviation: CI, confidence interval.

The pooling of data under the random-effects model is presented in Figure 6. The results show significant differences between the two groups, with Hedges’s g = −0.487 (95% CI: −0.646, −0.327) and *p* < 0.001, with effect sizes in individual studies ranging from -1.220 (95% CI: −1.551, −0.899, *p* < 0.001) [36] to 5.150 (95% CI: 4.172, 6.128, *p* < 0.001) [24]. Relative weights and standard residuals for each study are shown in Figure 6. Relative weights ranged from 1.98% [24] to 23.11% [36]. Standard residuals ranged from −4.95 [36] to 11.45 [24]. Heterogeneity was I^2^ = 96.267%, Q_7_ = 187.502, and τ^2^ = 1.423, *p* < 0.001, indicating high heterogeneity, which was higher than that found in hair and in whole blood.

**Figure 7:**
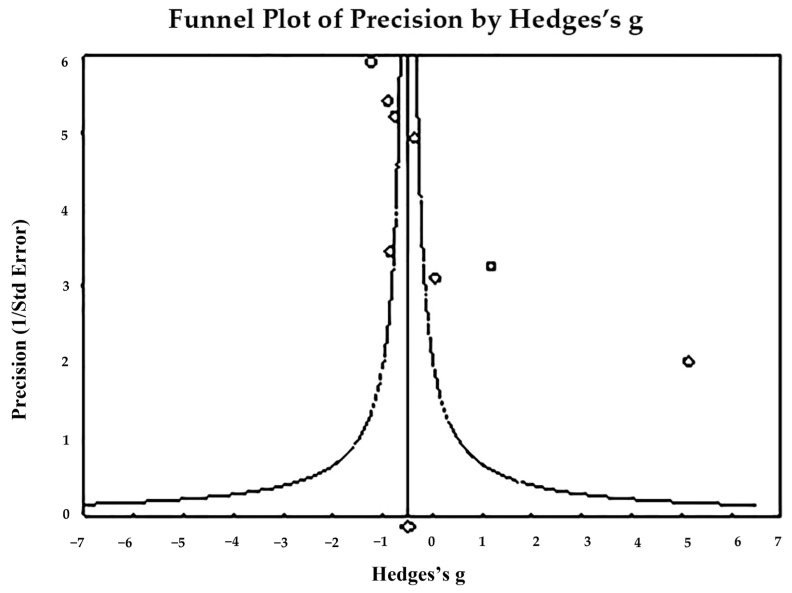
Funnel plot for assessment of publication bias in observational studies comparing urine Pb levels of controls and cases. The figure shows the studies’ effect sizes (Hedges’s g) relative to their precision (inverse of SE). Circles represent observed studies. Based on the observed studies, the diamond symbol indicates the pooled overall effect size. Publication Bias—Egger’s regression test: t_8_ = 4.225, *p* = 0.0056. Begg and Mazumdar Rank Correlation—Kendall’s τ = −0.857, *p* = 0.003.

The funnel plot (Figure 7) indicates publication bias—Egger’s regression test: t_8_ = 4.225, *p* = 0.0056; and Begg and Mazumdar rank correlation: Kendall’s τ = −0.857, *p* = 0.003. In conclusion, the pooling size effect shows significantly higher urine Pb levels in cases than in controls (*p* < 0.001).

In all three types of biological materials, we obtained high heterogeneity between the true mean effects (urine > hair > whole blood). This implies that practically all of the observed variability was due to real differences measured in the studies.

## 4. Discussion

It is suspected that autistic children have a reduced ability to excrete Pb and other toxic trace metals, leading to a high body burden and accumulation in various organs, including the central nervous system [34,75,76]. Disrupted Pb detoxification and excretion pathways are mostly connected to glutathione conjugation, which is often reduced in children with ASD [34]. Details of Pb’s deleterious effects on the nervous system, including the impaired release of calcium-dependent neurotransmitters, altered levels of essential zinc, oxidative stress, epigenetic changes, and other pathophysiological processes, are discussed elsewhere [4,36,77,78]. Herein, it is most important to emphasize that exposure to Pb begins prenatally, considering that this metal easily crosses the placental barrier and affects the blood–brain barrier, which is not yet developed until the first year [7,79,80,81,82].

### 4.1. Pb in Hair

In 1989, the Environmental Protection Agency (EPA) stated that hair is a meaningful and representative tissue for the quantification of toxic metals [83]. The National Health and Nutrition Examination Survey (NHANES) program and other programs around the world further bolstered the EPA’s claim and presented multiple studies on toxic metal levels in hair [84] and other human biological materials [85]. Hair grows at a rate of 1–1.5 cm per month and thus provides information on long-term exposure to Pb relative to body fluids [54]. Hair Pb profiling, according to Ambeskovic et al. [86], could provide a non-invasive and cost-effective health screening tool with predictive and diagnostic purposes, allowing risk assessment for vulnerable mothers and their offspring.

Most studies reported higher levels of Pb in the hair of case children than in controls, in Saudi Arabia [47,73], Oman [48], Iraq [19], Italy [49,58], Poland [32], Egypt [50,57], China [51], the USA [54], Kuwait [55], India [56], and Jordan [61]. Other studies reported lower levels of Pb in the hair of cases than in controls, including in Morocco [52], Russia [53], Korea [59], and the USA [60]. Details of the participants, analytical techniques used, and hair Pb level values are given in Table 1.

According to some studies, hair Pb levels can differ based on demographic or environmental factors. Thus, Mohamed et al. [50] reported that the hair Pb level in cases was positively correlated with living near gas stations (positive: 7.29 ± 7.41 µg/g vs. negative: 2.88 ± 4.02 µg/g), while no difference was found between age groups (2.98 ± 4.12 µg/g in <6 years old vs. 3.24 ± 4.52 µg/g in ≥6 years old). Ouisselsat et al. [52] found significantly higher hair Pb levels in girls with autism (1.15 µg/g) than in boys with autism (0.78 µg/g) (so, in girls, Pb was 32% higher). Rashaid et al. [61] reported that boys with autism had higher levels of Pb (19.48 ± 18.33) than control boys (5.96 ± 3.66 µg/g) and that girls with autism had higher levels of Pb (22.0 ± 11.7) than girls from the control group (8.01 ± 3.88 µg/g). They also reported that first-born cases had higher Pb levels compared to first-born neurotypical children. Skalny et al. [87] reported that the median hair Pb level in 16 samples from children between 3 and 4 years of age was numerically slightly higher (0.72 µg/g) than in 16 age-matched controls (0.60 µg/g), but without statistical significance. Cases aged 5–8 years had slightly higher hair median Pb levels (0.79 µg/g) than age-matched controls (0.46 µg/g) [87]. Zhai et al. [51] found that boys with autism had significantly more Pb in hair (1.85 µg/g) than neurotypical boys (0.80 µg/g). Furthermore, girls with autism had significantly higher Pb in hair (2.65 µg/g) than neurotypical girls (1.20 µg/g). Due to the variable results, additional research in this direction is needed.

We would like to point out that some studies were not included in our meta-analysis since they lacked a control group. However, these studies primarily looked at hair Pb levels and autism severity. For example, Fiore et al. [88] analyzed Pb levels in hair samples collected from 48 cases (boys/girls = 34/14, mean age = 6.5 ± 3.8 years). All participants were from Italy. They reported a median Pb level in hair of 0.54 µg/g. In addition, they found a positive correlation between hair Pb levels and the severity of autism symptoms (social communication deficits and repetitive, restrictive behavior). Furthermore, hair Pb levels were inversely correlated with IQ. In contrast, Geier et al. [89] enrolled 18 cases from Texas, USA and found no significant correlation between hair Pb levels and autism severity.

Three studies were excluded from our meta-analysis owing to a lack of numerical data, an insufficient control group, or an unsubstantiated diagnosis of autism. For example, Harchaoui et al. [90] did not offer SD values and so this study was excluded. Zhou et al. [91] compared Pb levels in hair from 50 autistic children with 50 sex- and age-matched cerebral palsy children. They found a numerically higher median Pb level in cases (1.87 µg/g) than in the cerebral palsy group (1.18 µg/g), but without statistical significance. Contrarily, they found significantly higher hair Pb in 34 cases (1.49 µg/g) than in the same age group of 30 children with cerebral palsy (0.69 µg/g). Ambeskovic et al. [86] analyzed Pb in hair collected from 75 children whose mothers experienced a natural disaster (mid-January 2011, Queensland Flood, Australia) during pregnancy. They did not find any association between this maternal prenatal stress and altered Pb levels in 4-year-old children. Furthermore, they reported that hair Pb levels were not associated with behavioral outcomes in children.

The findings from our current meta-analysis study, showing higher hair Pb levels in autistic children than in controls, did not accord with the previous meta-analysis by Guo et al. [92]. This can be attributed to the fact that Guo et al. [92] utilized data published from 1983 to 2017. These authors also verified that changes in analytical methodologies might have skewed the meta-results. Our meta-analysis of data for hair Pb levels, on the other hand, agreed with the recent meta-analysis results reported by Nakhaee et al. [40].

### 4.2. Pb in Whole Blood

Since Pb has a half-life in the circulation of weeks to months, it swiftly departs from the blood and accumulates in the bones and other organs. Because biopsies of these tissues are invasive, the assessment of Pb exposure in autistic people must be considered in available clinical samples [34].

Most studies reported higher Pb levels in the whole blood of autistic children than in neurotypical controls, including in the USA [34], Slovenia [62], China [44,68], Egypt [66], Nigeria [67], Tunisia [20], Syria [31], and Iraq [69]. One study reported lower Pb levels in the whole blood of cases than in controls [65], while, in other studies, Pb levels in cases and controls were numerically very similar [58,63,64]. Details of the participants, analytical techniques used, and whole blood Pb levels are given in Table 1.

Changes in whole blood Pb levels in autistic children according to sex, age, and other demographic factors have not been elucidated so far. An exception is the study of Hawari et al. [31], who found that Pb levels were significantly higher in cases aged 5 years or less (3.29 ± 0.29) than in controls (2.17 ± 0.65 µg/dL), and that male cases (4.6 ± 5.0) had higher Pb levels than female cases (2.07 ± 0.46 µg/dL).

One study was not included in our meta-analysis since there was no control group [93], while others lacked numerical data, such as the studies by Rahbar et al. [94] with Jamaican children and Rahbar et al. [95] with Pakistani children.

The results of this study were in agreement with Saghazadeh and Rezaei [35] and Shiani et al. [96], who conducted meta-analyses for blood Pb levels and reported significantly higher levels in cases than in controls.

In addition to the whole blood, Pb was also analyzed in RBCs. El-Ansary et al. [97] reported significantly higher Pb levels in the RBCs of 35 cases (6.04 ± 1.11 µg/dL) than in 30 controls (3.89 ± 0.88 µg/dL) (55% higher). They also found a negative correlation between Pb and selenium (Se) (r = −0.67) and indicated an antagonistic effect between these two elements. Adams et al. [34] also found significantly higher RBC Pb levels in cases than in controls (41% higher). However, these findings are not sufficient to conduct a meta-analysis of RBC Pb levels in autistic children.

### 4.3. Pb in Liquid Blood Fractions

Five of the six studies reported higher serum/plasma Pb levels in case children than in neurotypical controls [20,44,62,67,98]. Only Zhang et al. [99] reported numerically lower plasma Pb levels in cases than in controls, but without statistical significance. Interestingly, Dong et al. [100] divided cases into mild (n = 249, of which 208 were boys and 41 were girls) and moderate/severe symptoms (n = 263, of which 209 were boys and 54 were girls) and reported that higher serum Pb levels were significantly associated with the autism severity (2.58 ± 1.08 µg/dL in the mild group and 3.25 ± 1.89 µg/dL in the moderate/severe group). The number of papers that looked at Pb levels in the liquid blood fractions of cases and controls was insufficient to conduct a reliable meta-analysis due to several shortcomings, including the use of EDTA vacuum tubes, which can confound Pb levels.

### 4.4. Pb in Urine

Total urine Pb levels reflect approximately two thirds of the blood filtered by the kidneys during the last few days to weeks before sampling. Urine Pb levels, on the other hand, could offer information on long-term exposure if assessed in relation to bone turnover rates (slow—from the compact bone structure; and fast—from the trabecular bone structure) and the release of Pb from the bone into the circulatory system. Because bone remodeling happens more often in youngsters than in adults, these effects are unmistakably strong in younger people [30]. Higher urine Pb levels suggest increased exposure, increased absorption, higher body burden, and/or possibly decreased fecal excretion [70].

Table 1 contains information about the participants, the analytical techniques employed, and the urine Pb levels. Most studies reported higher levels of Pb in the urine of case children than in controls, including in the USA [34,70], Saudi Arabia [73], Egypt [74], and Pakistan [36]. In one study, the numerical values for urine Pb levels in cases and controls were nearly identical [58], whereas Pb levels in urine collected from Turkish children [71] and Spanish children [24] were lower in cases than in controls.

Some studies were not included in our meta-analysis due to several shortcomings. Abd Wahil et al. [30] reported a significantly lower mean urine Pb level in cases (0.26 ± 0.31 μg/dL) than in controls (0.58 ± 0.41 μg/dL), but cases and controls were not from the same place of residence in Malaysia. Rezaei et al. [101] reported significantly higher urine Pb levels in cases (6.19 ± 4.91 μg/dL) than in controls (2.11 ± 1.5 µg/L), but the authors claimed in one place in their paper that the results were presented as the median ± IQR and in another that the same results were presented as the median ± SD; therefore, this study was not clear to us and was not included in our meta-analysis.

### 4.5. Pb in Teeth

Lead was also analyzed in teeth. Abdullah et al. [80] used enamel regions of primary teeth from case children (n = 22) and controls (n = 22) to assess prenatal and early postnatal Pb exposure. However, they found no significant differences between prenatal Pb levels and postnatal Pb levels between the two examined groups. Adams et al. (2007) [102] also found no difference in Pb levels in primary teeth between cases (n = 16) and controls (n = 11). Although primary teeth can be very useful biomaterials for the assessment of cumulative Pb exposure during fetal development and early childhood, results for tooth Pb levels are insufficient to conduct a meta-analysis and draw meaningful conclusions.

## 5. Limitations

Although rigid criteria were used to select participants and exclude publications with very high Pb levels in biological materials, we were unable to segregate individuals based on sex, age, or country, because the dimensionality required for a meta-analysis was lost. However, in terms of Pb levels, the participant’s country and micro-location of residence could be a crucial aspect that should be studied further. As a result of our meta-analysis, we believe that more research on larger numbers of individuals with properly sex-, age-, and residence-matched controls and a wider range of biological materials is required.

## 6. Conclusions

Data from this systematic review and meta-analysis revealed that, compared with control children, autistic children had higher Pb levels in hair, whole blood, and urine, indicating a high body burden of this heavy metal. As a result, Pb exposure could be a risk factor in the genesis of autism, or other, unknown mechanisms could be at play that explain this difference. Since no Pb level can be considered safe, the data from this study undoubtedly point to the importance of monitoring Pb levels in autistic children in the future, namely in hair, whole blood or its parts (serum/plasma, RBCs), and urine. Other biological materials, such as teeth, appear to be promising. Additional research is needed to shed light on the reliable reduction of Pb levels in the bodies of children with autism and, thereby, reduce or prevent the harmful effects. Overall, we hope that the information given here will aid in the planning of future research on the adverse consequences of environmental Pb exposure and a better understanding of its involvement in autism.

## Figures and Tables

**Table 1:** Characteristics of 17 studies (2005–2023) on hair Pb levels (µg/g), 13 studies (2011–2023) on blood Pb levels (µg/L), and 8 studies (2010–2020) on urine Pb levels (µg/g creatinine) in controls and cases.

	Study	Country	Sample Size C/Cases	Age C/Cases	Sex (Girls/Boys) C/Cases	Analytical Technique	Pb Level (Mean ± SD) C/Cases
Hair							
1.	Al-Ayadhi, 2005 [47]	Saudi Arabia	80/65	7.2 ± 0.7/9 ± 0.3	C not specified; 4/61	AAS	0.96 ± 1.77/3.48 ± 1.43
2.	Al-Farsi et al., 2013 [48]	Oman	27/27	5.5/5.3	7/20; 2/22	ICP-MS	0.23 ± 0.56/0.45 ± 0.77
3.	Aljumaili et al., 2021 [19]	Iraq	20/75	3–14/3–14	Not specified	AAS	1.25 ± 0.66/3.44 ± 2.93
4.	De Palma et al., 2012 [49]	Italy	61/44	8.4 ± 3.1/9.0 ± 4.0	36/27; 7/37	ICP-MS	0.013 ± 1.57/0.029 ± 2.24
5.	Filon et al., 2020 [32]	Poland	30/30	5.09 ± 1.21/5.25 ± 1.59	5/25; 5/25	EDX	3.41 ± 1.21/6.03 ± 0.69
6.	Mohamed et al., 2015 [50]	Egypt	100/100	6.80 ± 3.04/6.20 ± 2.40	26/74; 16/84	AAS	2.06 ± 2.45/3.31 ± 3.92
7.	Zhai et al., 2019 [51]	China	58/78	4.90 ± 0.97/4.96 ± 1.01	27/31; 22/56	ICP-MS	0.86 ± 1.79/2.00 ± 3.25
8.	Ouisselsat et al., 2023 [52]	Morrocco	120/107	6.68 ± 2.39/7.14 ± 2.47	36/84; 25/82	ICP-MS	0.89 ± 0.65/0.72 ± 0.79
9.	Skalny et al., 2017a [53]	Russia	74/74	5.11 ± 2.34/5.12 ± 2.36	Not specified	ICP-MS	0.59 ± 0.37/0.51 ± 0.49
10.	Adams et al., 2006 [54]	USA	40/51	7.5 ± 3/7.1 ± 3	10/30; 12/39	ICP-MS	0.60 ± 0.52/0.89 ± 1.68
11.	Fido and Al-Saad, 2005 [55]	Kuwait	40/40	4.3 ± 2.6/4.2 ± 2.2	Only boys	ICP-MS	0.08 ± 5.07/0.17 ± 4.44
12.	Priya and Geetha, 2011 [56]	India	50/45	4–12	Not specified; 9/36	AAS	1.56 ± 0.18/3.24 ± 0.38
13.	Elsheshtawy et al., 2011 [57]	Egypt	32/32	4.0 ± 0.8/4.1 ± 0.8	8/24; 8/24	AAS	0.01 ± 0.01/0.07 ± 0.01
14.	Albizzati et al., 2012 [58]	Italy	20/17	10.41 ± 3.05/11.52 ± 3.2	6/14; 2/15	ICP-MS	0.28 ± 0.08/0.51 ± 0.22
15.	Jung et al., 2008 [59]	Korea	22/28	7.77 ± 1.50/7.63 ± 1.41	7/15; 9/19	ICP-MS	0.05 ± 0.04/0.04 ± 0.04
16.	Kern et al., 2007 [60]	USA	45/45	3.0 ± 1.4/3,00 ± 1.4	10/35; 10/35	ICP-MS	0.09 ± 0.08/0.06 ± 0.06
17.	Rashaid et al., 2021 [61]	Jordan	50/57	7.33 ± 2.37/7.60 ± 2.16	9/41; 11/46	ICP-MS	6.31 ± 3.74/19.95 ± 17.23
Whole blood							
1.	Adams et al., 2013 [34]	USA	44/55	11.0 ± 3.1/10.0 ± 3.1	5/39; 6/49	ICP-MS	8.80 ± 6.60/10.40 ± 9.80
2.	Macedoni-Lukšić et al., 2015 [62]	Slovenia	22/52	6.6. ± 3.7/6.2 ± 3.0	11/11; 6/46	AAS	18.60 ± 7.24/30.80 ± 34.33
3.	Li et al., 2018 [63]	China	184/180	6.12 ± 1.69/5.06 ± 1.37	38/146; 30/150	AAS	58.83 ± 18.92/56.82 ± 24.43
4.	Qin et al., 2018 [44]	China	38/34	4.29 ± 1.73/4.10 ± 0.81	17/21; 14/20	ICP-MS	18.90 ± 12.80/31.30 ± 4.06
5.	Albizzati et al., 2012 [58]	Italy	20/17	10.41 ± 3.05/11.52 ± 3.20	6/14; 2/15	ICP-MS	10.10 ± 1.20/10.90 ± 1.60
6.	Tian et al., 2011 [64]	USA	15/37	3.43 ± 0.50/3.68 ± 0.83	4/11; 5/32	ICP-MS	13.00 ± 5.80/13.00 ± 10.10
7.	Rahbar et al., 2015 [65]	Jamaica	100/100	2–8/2–8	Not specified	ICP-MS	27.30 ± 18.50/22.50 ± 22.30
8.	Yassa, 2014 [66]	Egypt	45/45	12.40 ± 2.04/11.30 ± 1.02	31/14; 32/13	ICP-MS	9.75 ± 5.80/55.24 ± 10.02
9.	Omotosho et al., 2021 [67]	Nigeria	25/25	6.18 ± 2.26 5.26 ± 1.45	Not specified	ICP-MS	54.30 ± 12.00/94.90 ± 20.20
10.	Zhao et al., 2023 [68]	China	30/30	4.2 ± 1.5/3.8 ± 1.3	15/15; 9/21	ICP-MS	11.13 ± 12.50/14.96 ± 9.63
11.	Chehbani et al., 2020 [20]	Tunisia	70/89	7.81 ± 3.41/7.53 ± 3.02	29/41; 15/74	AAS	1.59 ± 1.69/2.24 ± 3.15
12.	Hawari et al., 2020 [31]	Syria	30/31	6–12/6–12	10/20; 5/26	AAS	28.40 ± 7.00/41.91 ± 46.80
13.	Alawad et al., 2019 [69]	Iraq	47/60	4.37 ± 0.25	11/36; 16/44	AAS	14.27 ± 1.57/17.38 ± 1.86
Urine							
1.	Adams et al., 2013 [34]	USA	44/54	11.0 ± 3.1/10.0 ± 3.1	5/39; 6/49	ICP-MS	0.32 ± 0.45/0.57 ± 0.86
2.	Adams et al., 2017 [70]	USA	50/67	12.2 ± 7.5/11.5 ± 8.5	9/41; 12/55	ICP-MS	0.35 ± 0.38/0.59 ± 0.26
3.	Yorbik et al., 2010 [71]	Turkey	20/30	5.6 ± 0.5/6.9 ± 2.7	7/13; 6/24	ICP-MS	4.63 ± 3.83/1.19 ± 1.98
4.	Fuentes-Albero et al., 2015 [72]	Spain	34/35	7.7 ± 0.9/7.4 ± 0.5	10/24; 10/25	AAS	1.32 ± 0.04/0.60 ± 0.19
5.	Albizzati et al., 2012 [58]	Italy	20/17	10.41 ± 3.05/11.52 ± 3.2	6/14; 2/15	ICP-MS	0.73 ± 0.29/0.71 ± 0.29
6.	Blaurock-Bush et al., 2011 [73]	Saudi Arabia	25/25	6.25 ± 2.31/5.29 ± 1.90	6/19; 3/22	ICP-MS	3.36 ± 4.11/8.45 ± 7.33
7.	Metwally et al., 2015 [74]	Egypt	75/55	4.02 ± 4.01	18/57; 16/39	ICP-MS	3.60 ± 1.04/12.47 ± 17.46
8.	Nabgha-e-Amen et al., 2020 [36]	Pakistan	76/90	9.8 ± 3.3	22/54; 20/70	ICP-MS	1.73 ± 1.22/3.76 ± 1.95

**Abbreviations:** C (Controls, neurotypical children); ICP-MS (inductively coupled plasma mass spectrometry); AAS (atomic absorption spectroscopy); EDX (energy-dispersive X-ray). Number of controls: hair: 869; blood: 670; urine: 344. Total number of neurotypical children: 1883. Number of cases: hair: 915; blood: 755; urine: 373. Total number of autistic people: 2043.

**Table 2:** Quality assessment of studies included in the meta-analysis for Pb levels in cases (based on the Newcastle–Ottawa scale).

Study	Selection				Comparability	Outcome		Score	
	Representativeness (1)	Size (2)	Non-Respondents (3)	Exposure Determination (4)	Design/ Analysis	Determination of Outcome	Statist. Test	For Sample Type Average	
Hair									
Al-Ayadhi, 2005 [47]	a	a	b	a	a	a	a	6	
Al-Farsi et al., 2013 [48]	a	a	b	a	a	a	a	6	
Aljumaili et al., 2021 [19]	a	a	c	a	a	a	a	5	
De Palma et al., 2012 [49]	a	a	a	a	a	a	a	7	
Filon et al., 2020 [32]	a	a	a	b	a	b	a	5	
Mohamed et al., 2015 [50]	a	a	a	a	a	a	a	7	
Zhai et al., 2019 [51]	a	a	a	a	a	a	a	7	
Ouisselsat et al., 2023 [52]	a	a	a	a	a	a	a	7	
Skalny et al., 2017a [53]	a	a	b	a	a	a	a	6	
Adams et al., 2006 [54]	a	a	a	a	a	a	a	7	
Fido and Al-Saad, 2005 [55]	a	a	b	a	b	a	a	5	
Priya and Geetha, 2011 [56]	a	a	c	a	a	a	a	5	
Elsheshtawy et al., 2011 [57]	a	a	a	a	a	a	a	7	
Albizzati et al., 2012 [58]	a	a	a	a	a	a	a	7	
Jung et al., 2008 [59]	a	a	a	a	a	a	a	7	
Kern et al., 2007 [60]	a	a	a	a	a	a	a	7	
Rashaid et al., 2021 [61]	a	a	a	a	a	a	a	7	6.35
Whole blood									
Adams et al., 2013 [34]	a	a	a	a	a	a	a	7	
Macedoni-Lukšić et al., 2015 [62]	a	a	a	a	a	a	a	7	
Li et al., 2018 [63]	a	a	a	a	a	a	a	7	
Qin et al., 2018 [44]	a	a	a	a	a	a	a	7	
Albizzati et al., 2012 [58]	a	a	a	a	a	a	a	7	
Tian et al., 2011 [64]	a	a	a	a	a	a	a	7	
Rahbar et al., 2015 [65]	a	a	c	a	a	a	a	5	
Yassa, 2014 [66]	a	a	a	a	a	a	a	7	
Omotosho et al., 2021 [67]	a	a	b	a	a	a	a	6	
Zhao et al., 2023 [68]	a	a	a	a	a	a	a	7	
Chehbani et al., 2020 [20]	a	a	a	a	a	a	a	7	
Hawari et al., 2020 [31]	a	a	b	a	a	a	a	6	
Alawad et al., 2019 [69]	a	a	b	a	a	a	a	6	6.62
Urine									
Adams et al., 2013 [34]	a	a	a	a	a	a	a	7	
Adams et al., 2017 [70]	a	a	a	a	a	a	a	7	
Yorbik et al., 2010 [71]	a	a	a	a	a	a	a	7	
Fuentes-Albero et al., 2015 [72]	a	a	a	a	a	a	a	7	
Albizzati et al., 2012 [58]	a	a	a	a	a	a	a	7	
Blaurock-Bush et al., 2011 [73]	a	a	a	a	a	a	a	7	
Metwally et al., 2015 [74]	a	a	b	a	a	a	a	6	
Nabgha-e-Amen et al., 2020 [36]	a	a	b	a	a	a	a	6	6.75
**6.57**									

Selection: (1) Representativeness of sample—a, truly representative of the average of the target population; b, somewhat representative of the average of the target population; c, selected group of users; d, no description of sampling strategy. (2) Sample size—a, satisfactory; b, not satisfactory. (3) Nonrespondents—a, comparability between respondent and nonrespondent characteristics is established and response rate is satisfactory; b, response rate is not satisfactory or comparability between respondents and nonrespondents is not satisfactory; c, no description of response rate or respondent and nonrespondent characteristics. (4) Exposure determination—a, validated measurement instrument; b, measurement instrument not validated but instrument is available or described; c, no description of measurement instrument. Comparability: (1) Comparability of subjects based on design or analysis—a, study controls for main factor; b, study controls for each additional factor. Outcome: (1) Determination of outcome—a, independent blind assessment; b, linkage of records; c, self-report; d, no description. (2) Statistical test—a, the statistical test used to analyze the data is clearly described and appropriate and the measurement of the association is presented, including confidence intervals and probability level (*p*-value); b, the statistical test is inappropriate, not described, or incomplete. Calculation: a = 1; b = from the maximum 7, 1 is subtracted; c = from the maximum 7, 2 is subtracted. Quality assessment was modified based on criteria as described by Nakhaee et al. (2023) [40].

**Table 3:** Number of studies with statistically significant differences in hair, whole blood, and urine Pb levels of controls and cases compared to the total number of studies included in the meta-analysis.

	Total Number of Analyzed Studies	Significantly Higher Pb Level in Cases than in Controls	Significantly Lower Pb Level in Cases than in Controls	No Statistically Significant Changes
Hair	17	9	2	6
Whole blood	13	3	/	10
Urine	8	4	2	2

## Data Availability

Data are available from the corresponding author upon reasonable request.
